# Bi-HPDO3A as a novel contrast agent for X-ray computed tomography

**DOI:** 10.1038/s41598-023-43031-y

**Published:** 2023-10-05

**Authors:** Rebecca Rizzo, Martina Capozza, Carla Carrera, Enzo Terreno

**Affiliations:** 1 Department of Molecular Biotechnology and Health Sciences, Molecular Imaging Centre, University of Torino, Via Nizza 52, 10126 Turin, Italy; 2 Institute of Biostructures and Bioimaging, National Research Council, Via Nizza 52, 10126 Turin, Italy

**Keywords:** Medical research, X-ray tomography

## Abstract

A new bismuth-based CT agent was synthesized through a facile synthesis strategy. The in vitro stability, toxicity and CT performance were evaluated. The in vivo imaging performance was investigated using three different doses (0.5, 1.2 and 5 mmol/kg) and the result obtained at 1.2 mmol/kg was compared with the clinically approved CT agent iopamidol at the same dosage.

## Introduction

**Figure 1:**
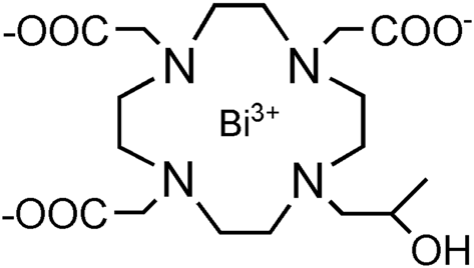
Bi-HPDO3A chemical structure.

Bismuth is a naturally monoisotopic (^209^Bi) element that is commonly considered the heaviest stable element of the Periodic Table due to the extremely long theoretical half-life of 1.9 × 10^9^ years^1^. Over the years, bismuth have found many applications in different fields starting from the preparation of non-toxic pigments and catalysts, the synthesis of biocompatible additives in dental materials^2^, and the development of therapeutics against *H. pylori* infections or in the treatments of ulcera gastritis and dyspepsia^3^. More recently, bismuth compounds have also been demonstrated active against coronavirus including both SARSCoV-1 and SARS-CoV-2, through the inhibition of a zinc enzyme helicase^4^. The biomedical applications of Bi were further implemented in the fields of imaging, cancer treatment, drug delivery and biosensing^5^. Beside the natural isotope ^209^Bi, the artificial ^212^Bi and ^213^Bi radioisotopes have been recently attracting interest as potential therapeutic radionuclides in targeted alpha therapy^6^. Especially, the nuclear properties of ^213^Bi are very suited for dosimetry studies and/or to monitor the biodistribution of the nuclide using single photon emission computed tomography (SPECT)^7^. However, Bi shows the highest X-ray absorption among the heavy metals at any energy of incident X-ray photons, thus making it a perfect candidate for designing X-ray Contrast Agents (XCAs)^8^. Moreover, Bi-compounds exhibit relatively low toxicity, when compared to other heavy metals^5^. Computed tomography (CT) has been widely employed in both medical and non-medical applications since the discovery of X-rays in 1895^9^. The technique relied on X-rays absorption by matter and the X-ray attenuation depends on tissues composition, in particular density and atomic number of the tissue components^10^. Whereas CT imaging of hard tissues such as bones and cartilages are very sensitives to X-rays, imaging of soft structures (fatty tissues or neoplastic formations) required contrast agents to improve the image performance^11^. Currently, the use of a CT agent requires the administration of the probe in the molar range, which represents one of the major downsides of this diagnostic tool compared to other imaging techniques such as MRI (sub-molar conc. range), nuclear and optical imaging (μmolar conc. range)^10^, especially in the field of molecular imaging. CT contrast agents are based on high-Z elements and may be classified in two major categories: small molecular agents (typically based on iodine or lanthanides) and nanoparticulate agents. Although iodine (Z = 53) has historically been the atom of choice for CT imaging applications, clinically employed iodinated contrast agents (e.g., iohexol, ioversol, and iopamidol) suffer from low sensitivity, poor spectral CT, potential allergy, and renal toxicity^12–14^. Hence, the development of improved CT contrast agents is still subject matter of interest. To date, the development of Bi-based CT agents is mostly focused on the design of nanoparticle systems because of their greater contrast density, longer circulation times and versatility for targeting purposes^11^. Representative examples are the Bi_2_S_3_ nanoparticles labelled with the cyclic nine amino acids peptide LyP-1 for targeting breast cancer^13^ and the MUC-16 aptamer targeted Bi-DOTA-PEG nanoparticles for imaging cervical cancer^14^. However, despite the promising preclinical results, no clinical translation has been reported so far for nanoparticulate agents, unlike small metal chelate-based contrast agents. On the other side, Bi-based small molecules have not been still sufficiently emphasized, with just a few examples reported, such as Bi-DPTA^15^ and Bi-DOTA^16^. On these premises, the aim of this study is to develop a new potential Bi-based small complex using the ligand HPDO3A (Fig. 1). HPDO3A is successfully used in MRI clinical exams since 2003, when its Gd(III) complex (Gadoteridol, marketed as ProHance^®^) received the approval for clinical use. A very important advantage of Bi-HPDO3A over the previously reported Bi-DOTA and Bi-DTPA complexes, is represented by its electroneutrality that may allow the administration of a higher dosage without affecting the osmolarity of the injected solution, and, as it has been clearly demonstrated for the complexation of lanthanide ions, without reducing the thermodynamic and kinetic stabilities of the metal complex^17^. The synthesis of Bi-HPDO3A is simple and cost-effective, and the ligand can be easily modified to allow the conjugation of the complex to targeting vectors^18^. In this work, the newly proposed agent has been characterized both in vitro and in vivo in terms of biocompatibility and CT imaging performance, using iopamidol as reference.

## Experimental section

### Chemicals

All chemicals were purchased from Sigma-Aldrich, all the solvents from VWR, HPDO3A and Iopamidol (Isovue 370) were kindly provided by Bracco Imaging S.p.A.

Electrospray ionization (ESI)-mass spectrometer (ESI-MS) of Bi-HPDO3A was obtained on Waters system (3100 Mass Detector, 2525 quaternary pump, 2767 sample manager, 2996 PDA detector). ^1^H-NMR spectra and ^13^C-NMR spectra were measured on a Bruker Avance spectrometer (600 MHz) instrument. Chemical shifts are reported in parts per million (ppm) and are referenced to tetramethylsilane.

UV/Vis spectrophotometric measures were performed on a UV/Vis spectrophotometer (6715, Jenway).

### Cell cultures and animals

Monocyte-macrophages (J774 cells) were purchased from ATCC. Cells were cultured until confluence using DMEM (Euroclone) medium supplemented with glutamine (2 mM), 10% fetal bovine serum (FBS, Sigma-Aldrich, St. Louis, MO, USA) and penicillin/streptomycin antibiotics (10,000 IU/mL penicillin, 10,000 IU/mL streptomycin, Corning Cellgro, Manassas, VA). MTT Cell Proliferation Kit was purchased from OZ Bioscience.

5-weeks-old BALB/C male mice were obtained from the animal facility of the Department of Molecular Biotechnology and Health Sciences (University of Turin, Italy). Animal manipulation and experimental procedures were carried out in accordance with the European Community guidelines (directive 2010/63) and under the approval of the Italian Ministry of Health (authorization #229/2016).

### Synthesis and characterization of Bi-HPDO3A

**Figure 2:**
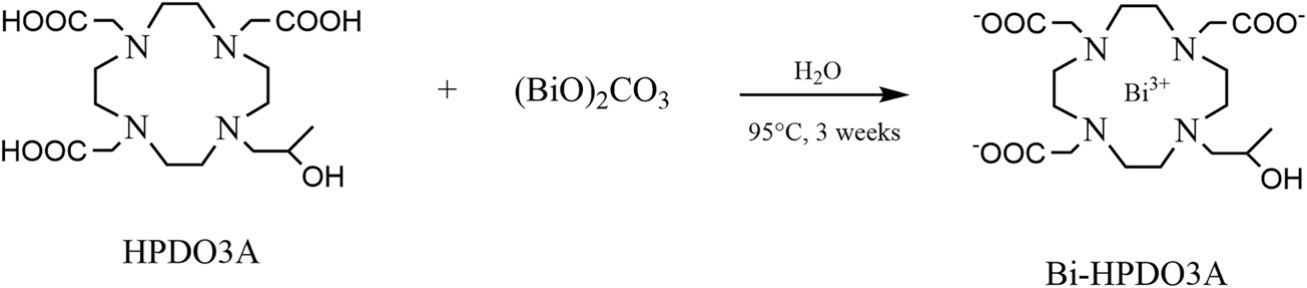
Bi-HPDO3A synthetic procedure.

Bi-HPDO3A complex was synthetized starting from HPDO3A ligand and bismuth(III) carbonate in a 2:1 molar ratio, respectively (Fig. 2). 1 g (2.36 mmol) of ligand was solubilized in 15 mL of deionized water prior to bismuth carbonate addition (0.75 g, 1.18 mmol) and the mixture was heated at 95 °C for 3 weeks. The xylenol orange test was performed to monitor the reaction progress and a small amount of ligand was added to have a free metal quantity not exceeding 0.1%. The reaction suspension was filtered (0.45 µm pore size) and the filtrated was lyophilized to obtain a white solid. The compound was then used without further purification.

The metal chelate was characterized by ^1^H-NMR, UV–Vis spectrophotometry, and ultra-performance liquid chromatography-mass spectrometry (UPLC-MS). The new bismuth agent was dissolved in deionized water and diluted to the concentration of 0.4 mM for UV/Vis measurements (200–500 nm range). For NMR characterization, the complex was dissolved in D_2_O to a final concentration of 3 mg/mL and, the ^1^H- and ^13^C-NMR spectra were acquired at room temperature. The complex was also characterized by ESI–MS, and UPLC-UV/Vis spectra were recorded to assess the purity.

### Stability measurements over time and transmetallation with Zn(II)

Physical stability of an aqueous solution of the complex was checked within 14 days to assess colloids formation. Stability in PBS and human serum at 37 °C was monitored within 24 h by measuring the absorbance of the Bi-complex (0.123 M) at 305 nm at 3 different times (3, 6 and 24 h). Transmetallation of Bi-HPDO3A towards Zn(II) ions was investigated by measuring the absorbance of the Bi-complex at 305 nm at 3 different times (3, 6 and 24 h) after mixing equimolar amount (20 mM) of Bi-HPDO3A and Zn(II) at 37 °C and pH 7.4.

### In vitro cytotoxicity

Cellular methyl thiazolyl tetrazolium (MTT) assay was carried out to evaluate the cytotoxicity of Bi-HPDO3A in vitro. J774 cells (50 k cells/well) were incubated in a 96-wells plate for 24 h. The medium was then replaced by fresh one containing different concentrations of Bi-HPDO3A (3, 6, 12.5, 25, 50, 125, 250, and 500 µM). After the incubation, cells were incubated with MTT reagent for 3 h. Then, the solubilization solution was added to dissolve the purple formazan crystals. After 30-min of mild shaking, the absorbance value at 600 nm of each well was recorded through a microplate reader. The experiment was conducted in triplicate in 3 different experimental sessions.

### CT imaging

Aqueous solutions at different Bi-HPDO3A concentrations (3–400 mM range) were prepared, and in vitro CT imaging was performed (MiLabs VECTor^6^, 65 kV, 0.25 mA, 45 ms). A commercially available solution of iopamidol (Isovue-370^®^ Bracco Imaging), properly diluted, was used as reference.

For in vivo CT imaging experiments, mice were anaesthetized with isoflurane (1.5–2.5%, 1 mL/min oxygen flow rate) and then scanned before and after the injection via the tail vein of 100 µL of Bi-HPDO3A or iopamidol solutions to reach a dose of 0.5, 1.2 and 5 mmol Bi/kg bw or 1.2 mmol I/kg bw, respectively. CT scans were acquired at different time points (0, 1 min, 5 min, 20 min, 40 min, 1 h). The parameters were set as follows: field of view 54 × 140 mm, tube current 0.25 mA, tube voltage 65 kV, exposure time 45 ms. VOIs analysis for kidneys and bladder were performed to semi-quantify the complex distribution in these main involved organs.

### Histology

To verify possible acute toxicity effects of Bi-HPDO3A on kidneys, hematoxylin–eosin (HE) staining was carried out on mice kidneys explanted 7 days and 14 days after the administration of the agent (5 mmol Bi/kg bw).

### Ethical approval

The study is reported in accordance with ARRIVE guidelines.

## Results and discussion

### Synthesis and characterization of Bi-HPDO3A

The Bi-HPDO3A complex was characterized by multiple analytical methods. The ^1^H-NMR spectra (600 MHz, D_2_O, pH 6.5, 298 K) of Bi-HPDO3A was characterized by the following signals (TMS as reference): 1.19 ppm (d, 3H, methyl group), 3.10–3.67 ppm (m, 19H, ethyl groups), 3.81 ppm (m, 3H, ethyl group), 4.05 ppm (d, 1H, hydroxyl), 4.36–4.49 ppm (m, 3H, ethyl group) (See Supplementary Information Fig. S3).

The ESI-MS spectrum of Bi-HPDO3A was characterized by m/z peaks found at 306.02, 611.16 and 1221.44, which correspond to [M+2H]^2+^, [M+H]^+^ and [2M+H]^+^, respectively. Furthermore, as calculated from the UPLC-UV/Vis spectrum, the metal complex was obtained with a purity > 99%. See Supplementary Information (Figs. S1, S2).

### In vitro stability

Colloidal stability of a solution of Bi-HPDO3A 0.123 M (1.2 mmol/kg) was checked within 14 days after storage at room temperature. The solution remained clear without any precipitation event after centrifugation at 4000 rpm for 10 min. Structural stability of the bismuth complex was investigated by means of UV–Vis measurements. The complex proved to remain intact both in PBS and HS for 24 h at 37 °C (Fig. S11). Crucial aspect to be considered for the potential clinical translation of a metal complex is to check the stability towards transmetallation. Due to the relatively high blood concentration (55–125 µM) and the high affinity to polyamino-polycarboxylic ligands, Zn(II) is typically used as competitor endogenous metal ion to predict the in vivo stability of metal complexes^19^.

**Figure 3:**
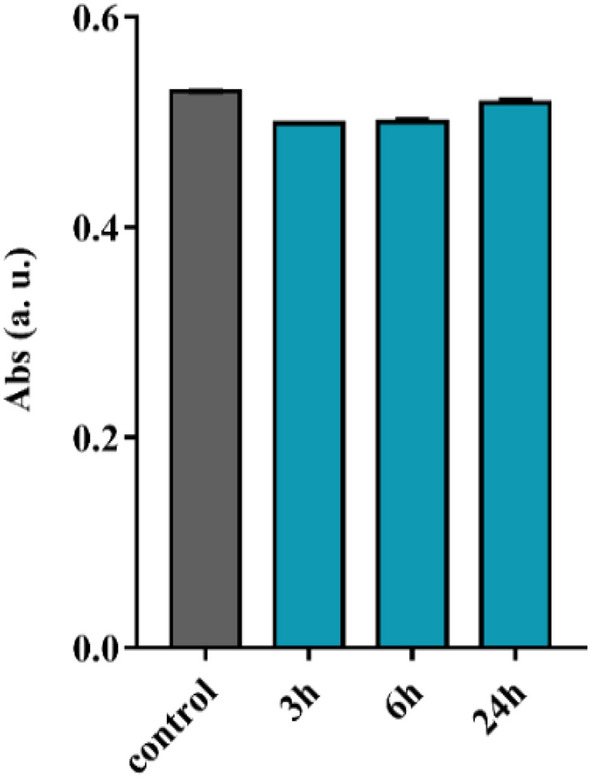
UV measurements at λ = 305 nm for transmetallation study of Bi-HPDO3A (20 mM) with Zn^2+^ (20 mM).

A calibration curve was preliminary assessed for Bi-HPDO3A (Fig. S4) and the whole UV–Vis spectrum was recorded to establish the maximum absorption wavelength (λ_max_, 305 nm) of the complex. Figure 3 indicates that the absorbance of Bi-HPDO3A at 305 nm in presence of an equimolar amount of Zn(II) ions was not affected after one day of incubation at 37 °C and pH 7.4. This result fully agrees with the high thermodynamic and kinetic stability expected for Bi-HPDO3A complex. In fact, it has been reported that Bi(III) forms octacoordinated highly stable complexes and log K_ML_ values of 30.3 and 26.8 have been reported for DOTA and an HPDO3A-like ligand, respectively^20^.

### Cytotoxicity

The cytotoxicity of Bi-HPDO3A was assessed by the standard MTT test on a murine cell line of macrophages (J774). Cells were incubated for 24 h with different concentrations of the Bi-complex (0–500 µM range).

**Figure 4:**
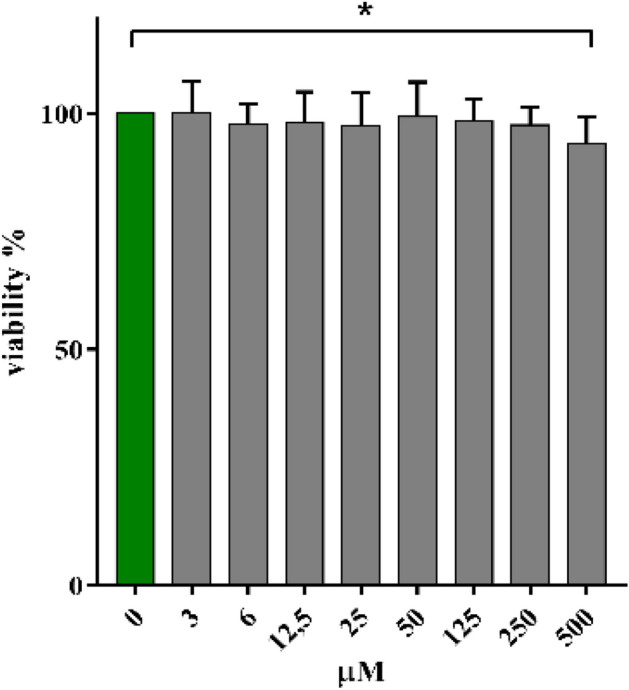
Viability % on J774 cells treated with Bi-HPDO3A (24 h, 0–500 µM). P* = 0.044.

The result of the MTT test (Fig. 4) revealed an excellent biocompatibility with cell viability values attesting from 90 to 100% with no statistically significant differences (statistical analyses were conducted using the one-way ANOVA method and statistical significance was defined at P values less than 0.05) in the examined concentration range except for the concentration of 500 µM.

### In vitro CT imaging performances

**Figure 5:**
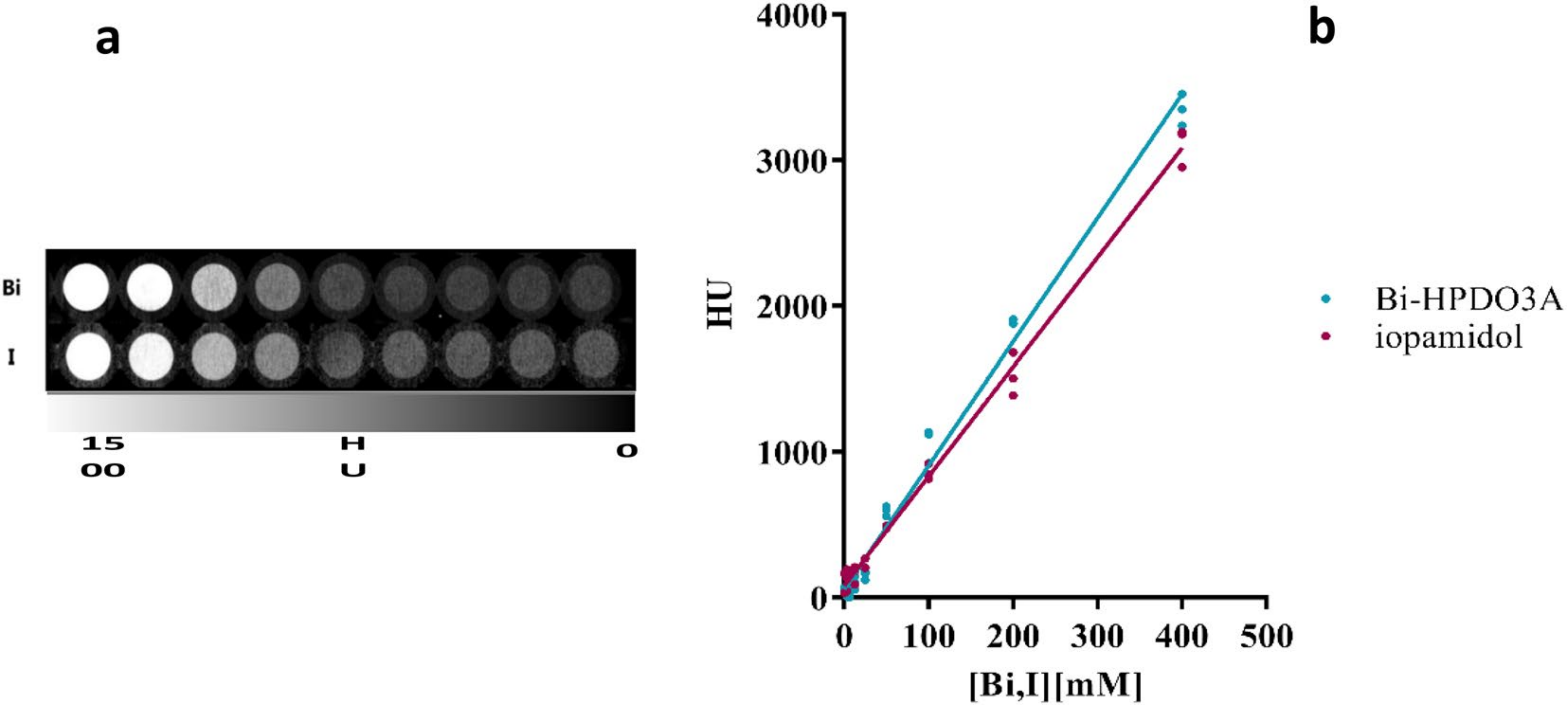
(**a**) Phantom CT imaging. (**b**) Comparison between Bi-HPDO3A and iopamidol in vitro CT attenuation capabilities.

The in vitro CT performance of Bi-HPDO3A was evaluated by measuring the X-ray attenuation as a function of the probe concentration in the 0–400 mM range (Fig. 5). Data were compared with solutions of iopamidol containing iodine concentrations in the same interval. The expected linear correlation between HU units and atom concentration was found for both agents. Unfortunately, the voltage limitations imposed by the available scanner (65 kV) prevented to exploit the larger X-ray attenuation coefficient of Bi at higher voltage, and in this acquisition setting, the two agents performed very similarly.

### In vivo CT imaging experiments and toxicity assessment

The promising properties observed in vitro for Bi-HPDO3A prompted us to preliminarily test the in vivo performance of the agent on healthy mice.

**Figure 6:**
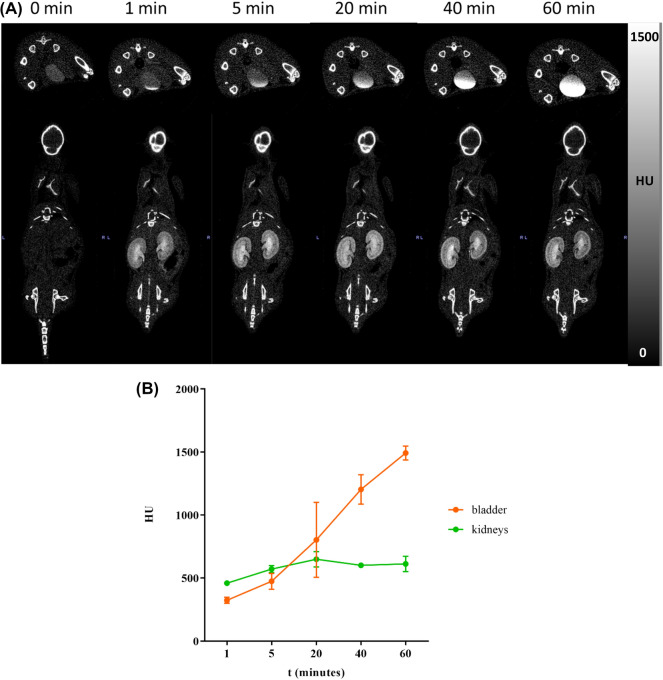
(**A**) In vivo CT imaging after *i.v.* administration of 5 mmol/kg of Bi-HPDO3A. Bladder and kidneys CT contrast are displayed. (**B**) VOIs analysis on bladder and kidneys for a dose of 5 mmol/kg of Bi-HPDO3A.

**Figure 7:**
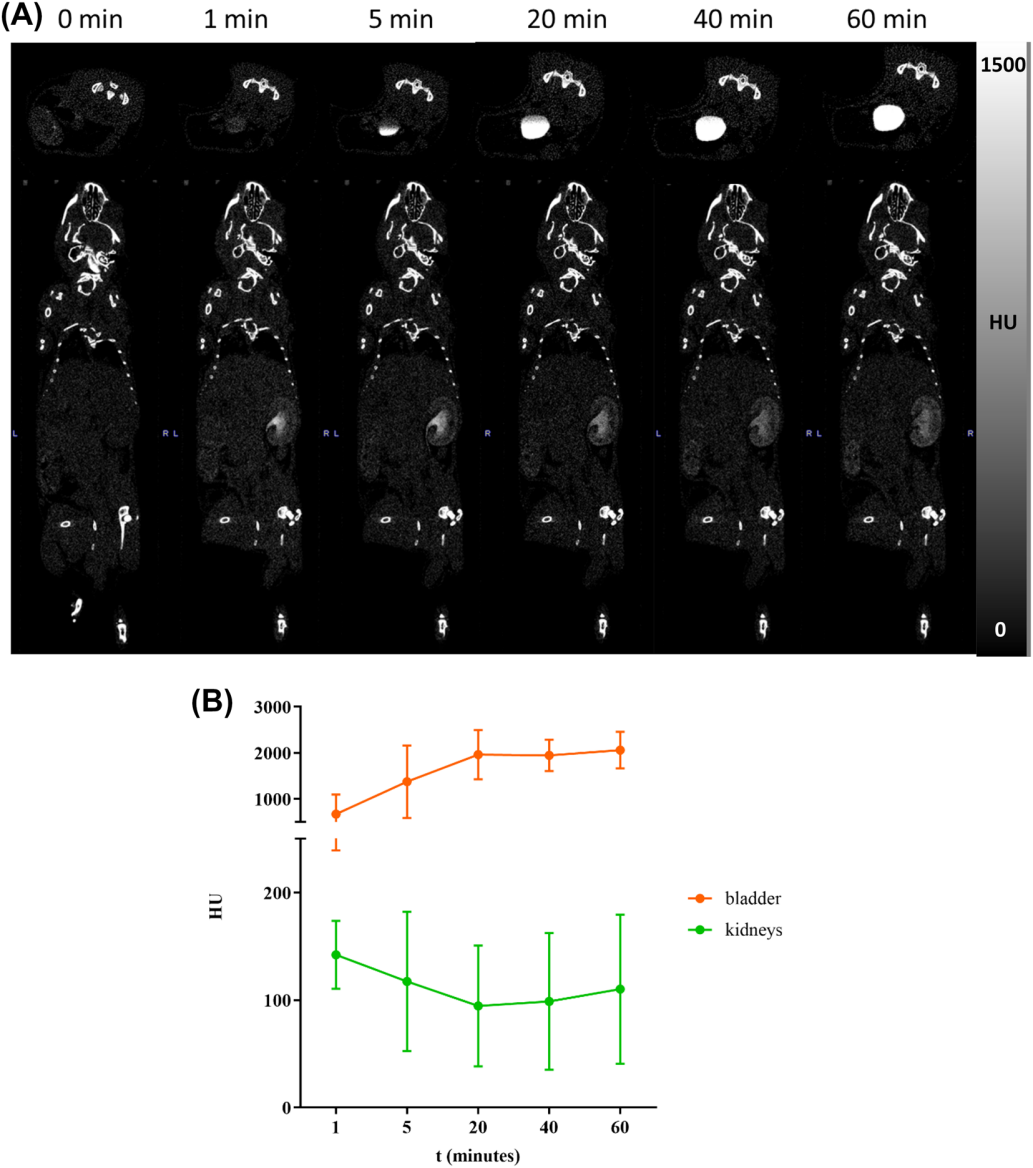
(**A**) In vivo CT imaging after i.v. administration of 1.2 mmol/kg of Bi-HPDO3A. Bladder and kidneys CT contrast are displayed. (**B**) VOIs analysis on bladder and kidneys for a dose of 1.2 mmol/kg of Bi-HPDO3A.

**Figure 8:**
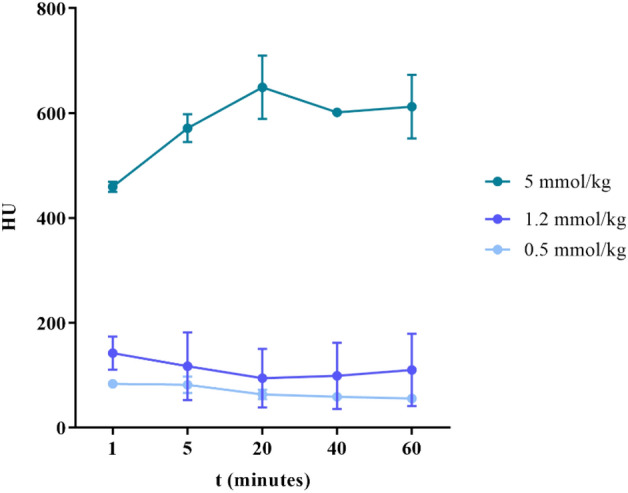
Dynamics of the kidney CT contrast for the three different injected doses of Bi-HPDO3A.

The first dosage of 5 mmol/kg of Bi-HPDO3A were intravenously injected into BALB/C mice to assess biodistribution and in vivo X-ray attenuation capability of the agent. A high CT contrast was observed both in kidneys and bladder (Fig. 6A,B). Kidneys were already lighted up 1-min post injection, and within 60 min the bladder CT contrast signal increased linearly. Interestingly, after a rapid increase, the enhancement of the renal signal remained largely unchanged after 20 min post injection. Encouraged from this positive result, a lower injected dose (1.2 mmol/kg) was tested. This value was calculated starting from the clinical injected dose of the MRI agent Gadoteridol, scaled down to mice, taking into account the difference in body surface area between the two organisms^21^. Despite the expected lower CT contrast, a clear signal was detected in the renal collecting system (calyx and ureter, Fig. 7A,B, Fig. S4). To further challenge the attenuation capability of the Bi-HPDO3A complex, a further dose reduction (down to 0.5 mmol/kg) was attempted (Fig. S6). Expectedly, the CT contrast was the lowest among the three conditions explored, and the signal decreased according to the dose (Fig. 8). The signal was only detected on the calyx portion of the collecting system with a maximum peak 5-min post injection to finally disappeared 40-min post injection.

For comparative purposes, the imaging performance of Bi-HPDO3A was challenged with the clinically approved iodine-based iopamidol, using the injected dose of 1.2 mmol/kg. Iopamidol allowed for the visualization of the whole renal region (Fig. S8a) and, when compared to Bi-HPDO3A, displayed a slower renal clearance (Fig. S8b) with the kidney contrast that progressively increased in the first hour post-injection, in agreement with literature data^22^.

The different contrast dynamics displayed by the two probes in the kidneys is reported in Fig. 8.

Considering that the amount of the injected probes was equal, the higher contrast detected for iopamidol is primarily due to the higher molar concentration of attenuating atoms (3 iodine atoms/molecule vs 1 Bi atom/molecule), whereas the different dynamics can be explained in terms of the different hydrophilicity of the two agents, with the more hydrophilic Bi-HPDO3A complex that showed a faster renal clearance. To preliminary investigate the in vivo acute toxicity of the new compound in the main excretory organ, HE staining of the kidneys of mice injected with the highest dose (5 mmol/kg) of agent was performed. No tissue-damaging effects were found (Fig. S10), thus confirming the good safety profile of the new probe.

## Conclusions

This study presents a novel bismuth-based potential CT contrast agent based on the HPDO3A ligand. The high X-ray attenuation coefficient of bismuth, its wide safety profile, and the well-known characteristics as chelating agent of HPDO3A, already clinically employed, makes it an excellent candidate for this purpose. Although the sub-optimal voltage (65 kV) of the available scanner, Bi-HPDO3A has proved to have a good potential as CT contrast agent for imaging kidneys and bladder. The high-water solubility of this agent led to a rapid renal excretion, faster than Iopamidol, thus reducing possible risks of toxicity caused by unwanted tissue accumulations. The results herein reported suggest further investigations. Multimeric versions of this ligand, or similar macrocyclic ligands forming neutral complexes with trivalent metals, have been already reported, which could improve detection sensitivity, in analogy to what has been done with iodinated agents^23,24^. Furthermore, HPDO3A ligand is suitable of conjugation with chemicals for modulating and optimizing the biodistribution of the agent, thus opening new potential applications, in addition to image renal diseases.

### Supplementary Information

Supplementary Information.

## Data Availability

Data will be made available upon request to Rebecca Rizzo.
