# Supplementary material for: Bi-HPDO3A as a novel contrast agent for X-ray computed tomography
Source: Sci Rep. 2023 Oct 5;13:16747. doi: 10.1038/s41598-023-43031-y (PMC10556142; doi:10.1038/s41598-023-43031-y)
Supplement: Supplementary file 1 — Supplementary Information. [file 41598_2023_43031_MOESM1_ESM.docx]

**Bi-HPDO3A as a novel contrast agent for X-ray computed tomography**

Rebecca Rizzo^1^, Martina Capozza^1^, Carla Carrera^2^, Enzo Terreno^1*^

^1^ Department of Molecular Biotechnology and Health Sciences, Molecular Imaging Centre, University of Torino, Via Nizza 52, 10126, Torino (Italy)

^2^ Institute of Biostructures and Bioimaging, National Research Council, Via Nizza 52, 10126, Torino (Italy)

**SUPPLEMENTARY INFORMATION**

**UPLC-UV/Vis-MS**

The chromatographic runs were performed using a Kinetex F5 column 100 x 2.1 mm, 1.7 µm (Phenomenex). The column oven was maintained at 25°C and the elution solvents were water/trifluoroacetic acid 0.1% (solvent A) and acetonitrile/trifluoroacetic acid 0.1% (solvent B). The gradient was isocratic at 99% A for 8 min, then the composition was varied by a linear gradient (A:B, v/v) from 99:1 to 0:100 in 2 min to elute possibly present hydrophobic compounds (retention time of Bi-HPDO3A 1.05 min, Fig. S2). The flow rate was 0.4 mL/min, and the total run time was 10 min.


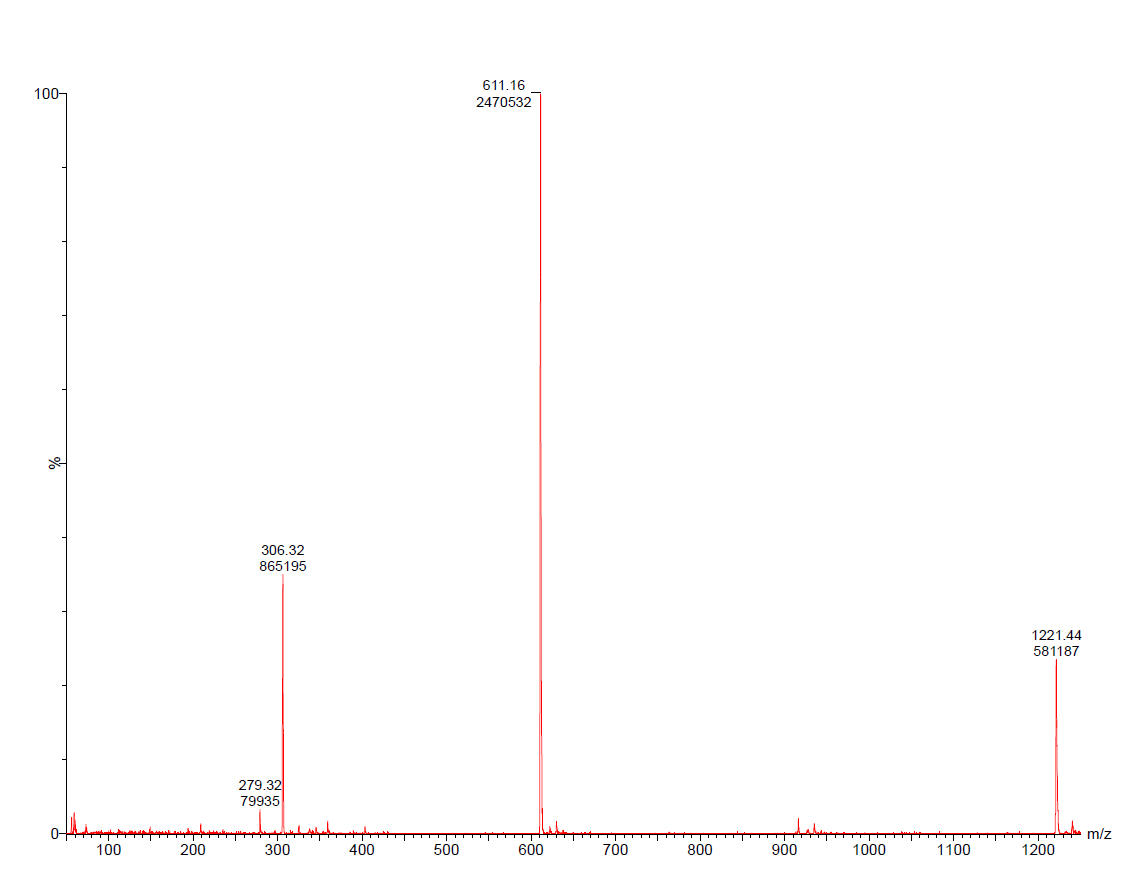
The high-resolution Waters 3100 Mass Detector was operated with an electrospray ion source in positive ion mode with a cone voltage of 20V. The intervals of mass scan were 50–1250 m/z. MS (ESI+): [2M+H]+ 1221.44 (obsd) 1221.44 (calcd); [M + H]+ 611.16 (obsd) 611.16 (calcd); [M + 2H]2+ 306.32 (obsd) 306.32 (calcd) (Figure S1).

**Figure S1** ESI-MS spectrum of Bi-HPDO3A. [2M-H]+ = 1221.44 m/z, [M-H]+ = 611.16 m/z, [M-2H]2+ = 306.02

UV/Vis spectra was recorded at 214 and 254 nm. The reported UV/Vis spectra (Figure S2) was recorded at 214 nm.


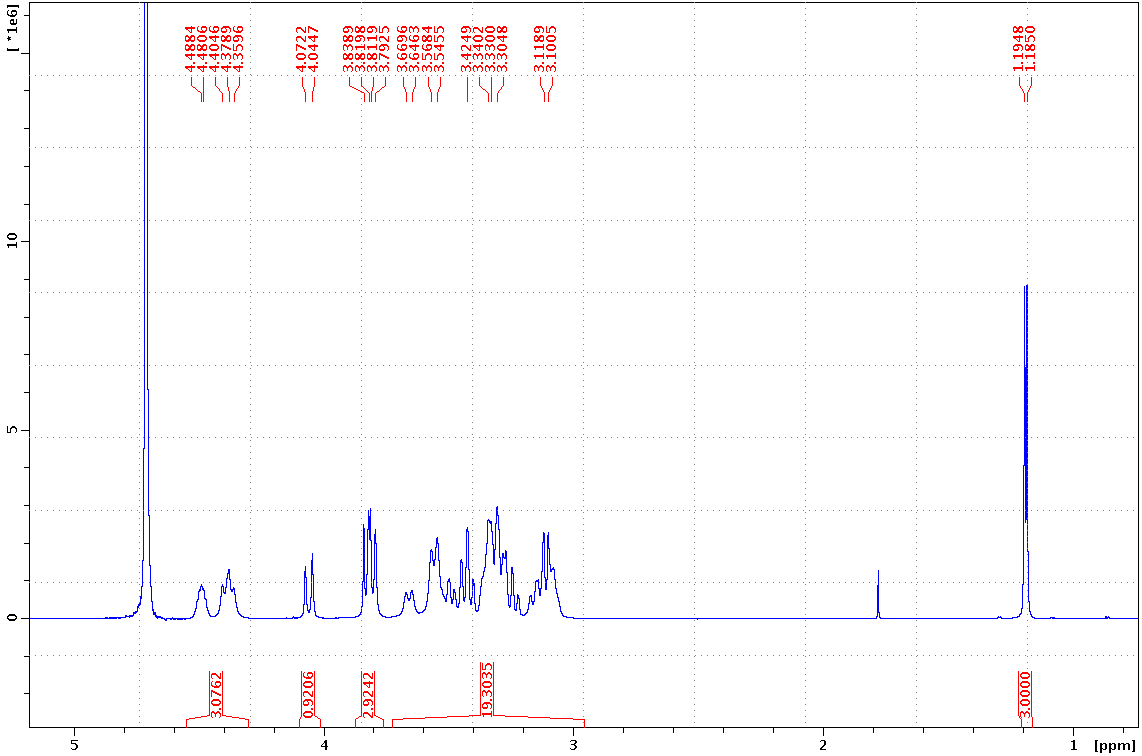

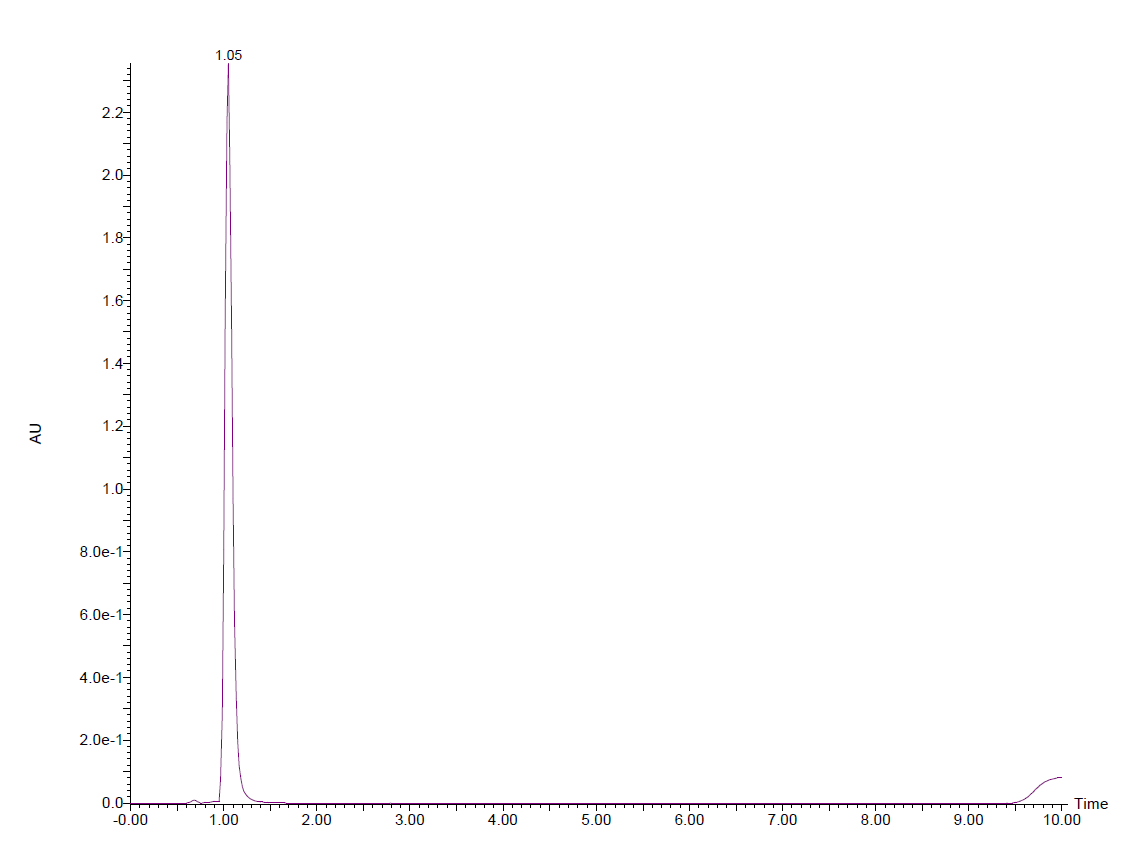


**Figure S3** H1-NMR (600 MHz) spectrum of Bi-HPDO3A in D_2_O (pH=6.5, T=298K). Chemicals shift (in ppm) and integrations are reported: δ 1.19 (d, 3H), δ 3.10-3.67 (m, 19H), δ 3.81 (m, 3H), δ 4.05 (d, 1H), δ 4.36-4.49 (m, 3H).

**Figure S2** UPLC-UV/Vis spectrum at 214 nm of Bi-HPDO3A.


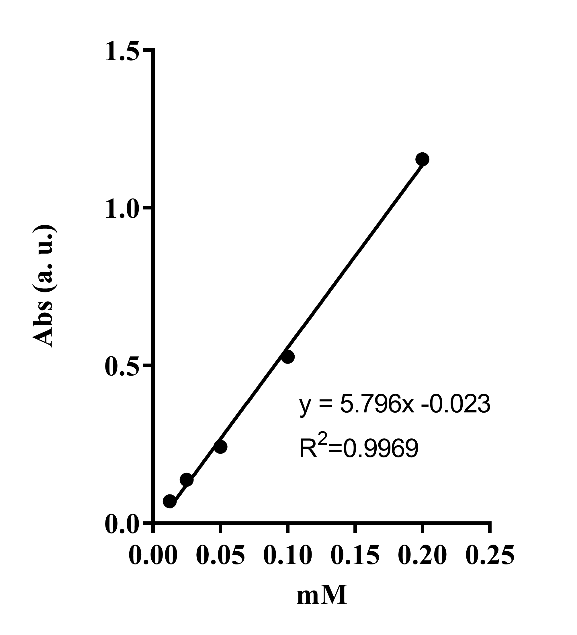

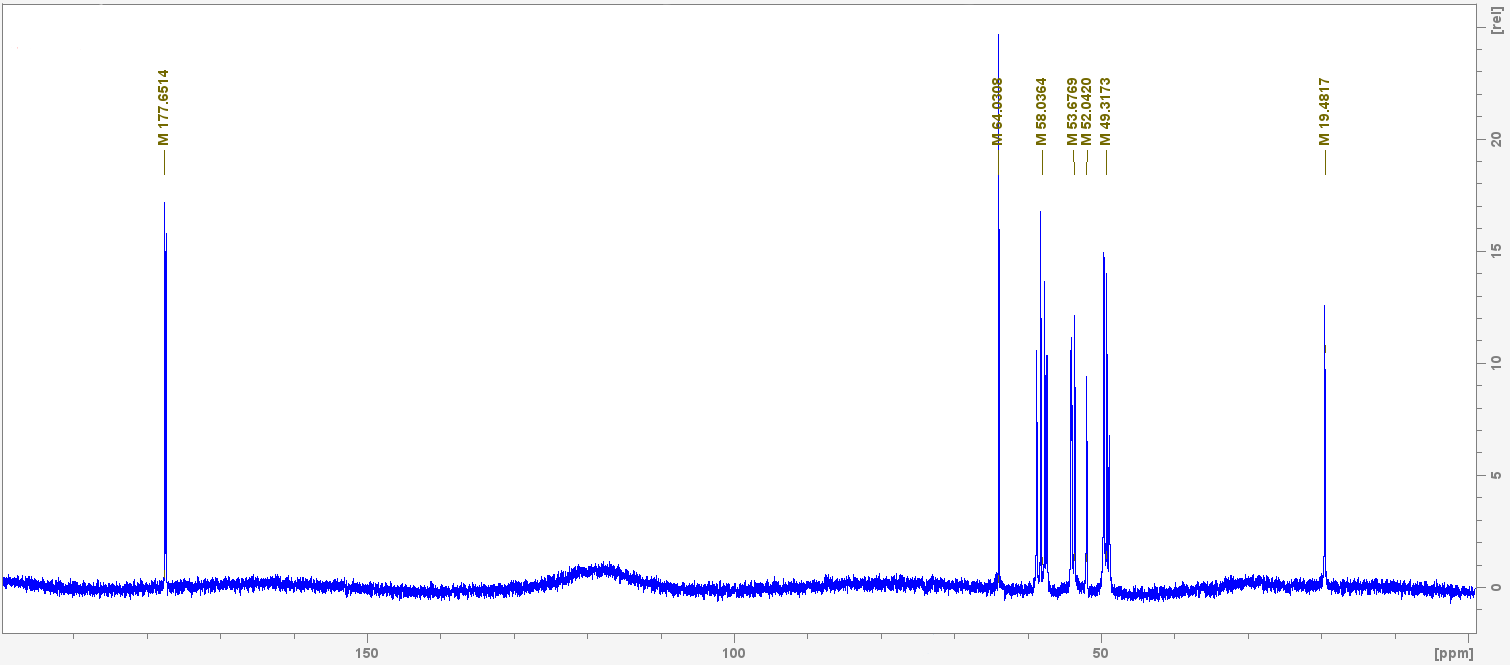


**Figure S4** 13C-NMR (600 MHz) spectrum of Bi-HPDO3A in D_2_O (pH=6.5, T=298K). Chemicals shift (in ppm) are reported: δ 19.48, δ 49.32, δ 52.04-53.68, δ 58.04, δ 64.04, δ 177.65.

**Figure S5** Calibration curve of Bi-HPDO3A at 305 nm for transmetallation study.


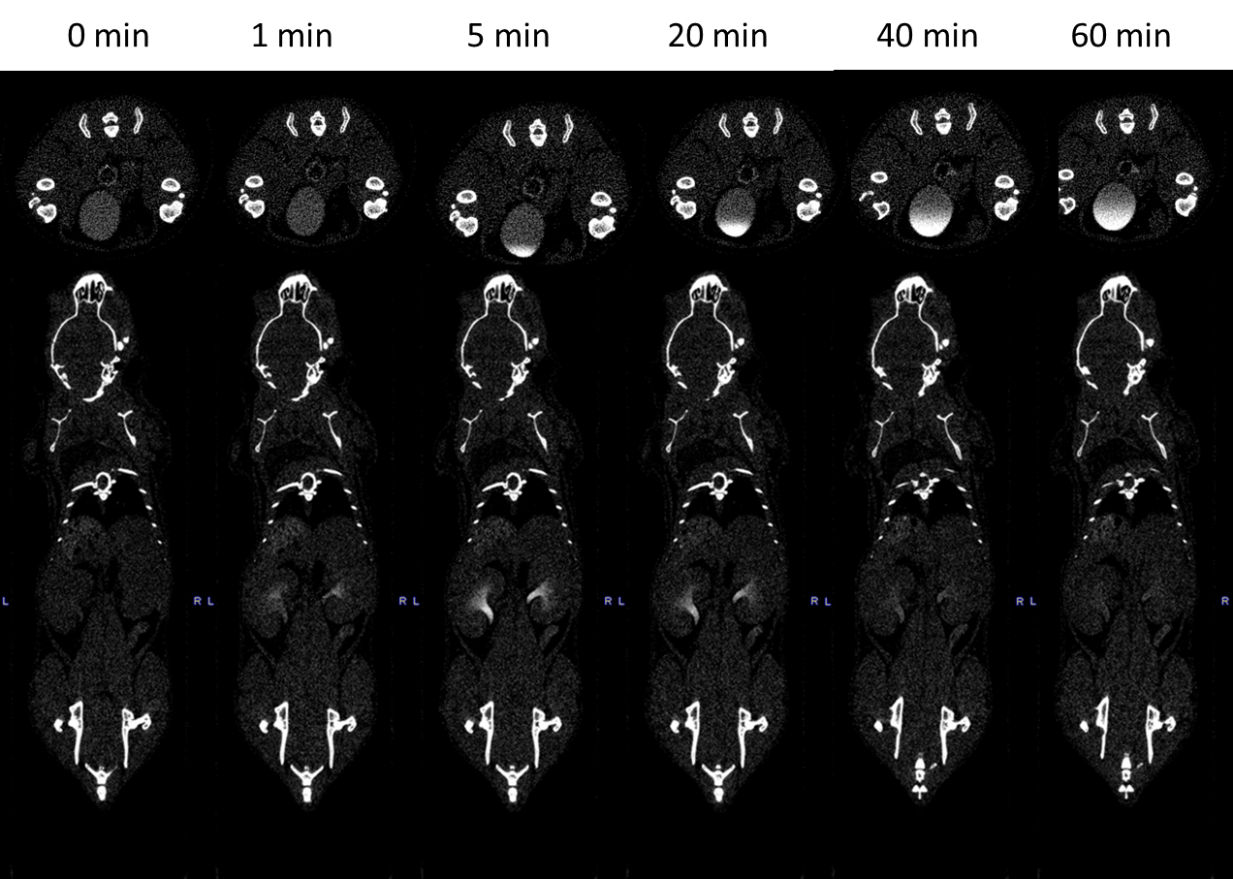


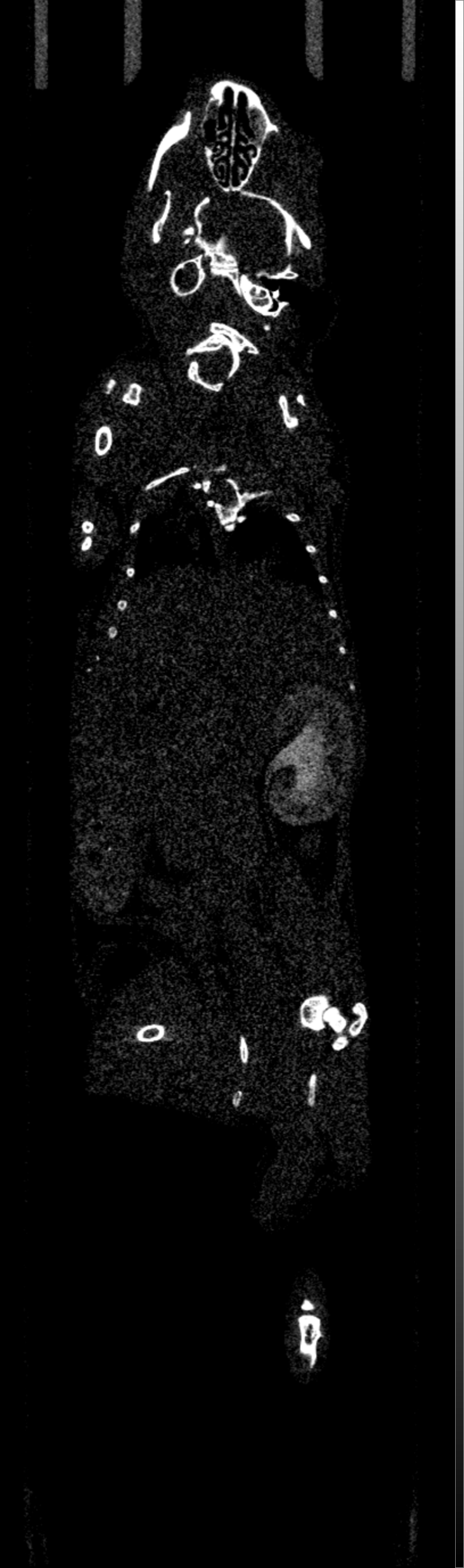


**1500**

**HU**

**0**

**Figure S6** *In vivo* CT imaging after *i.v.* administration of 0.5 mmol/kg of Bi-HPDO3A. Bladder and kidneys CT contrast are displayed.


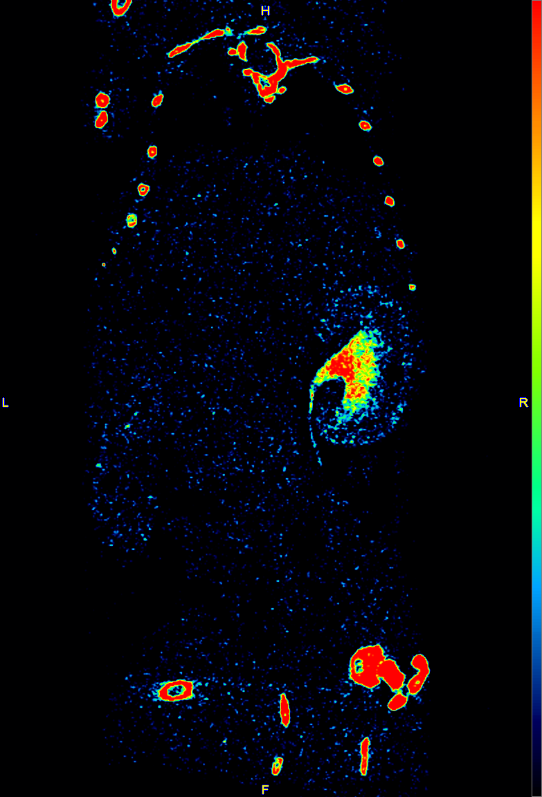

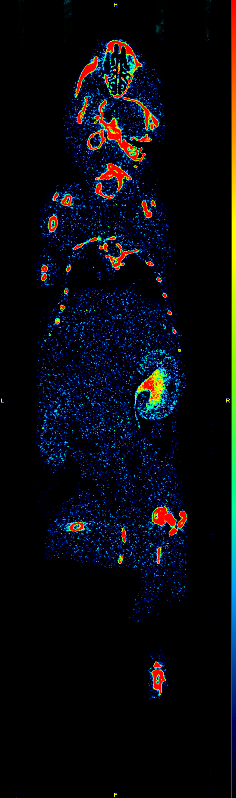


**Figure S7** Ureter CT enhancement after *i.v.* administration of Bi-HPDO3A 1.2 mmol/kg (5 min).

**1500**

**HU**

**0**


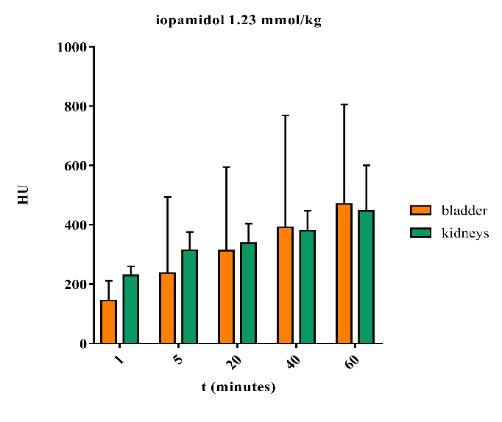

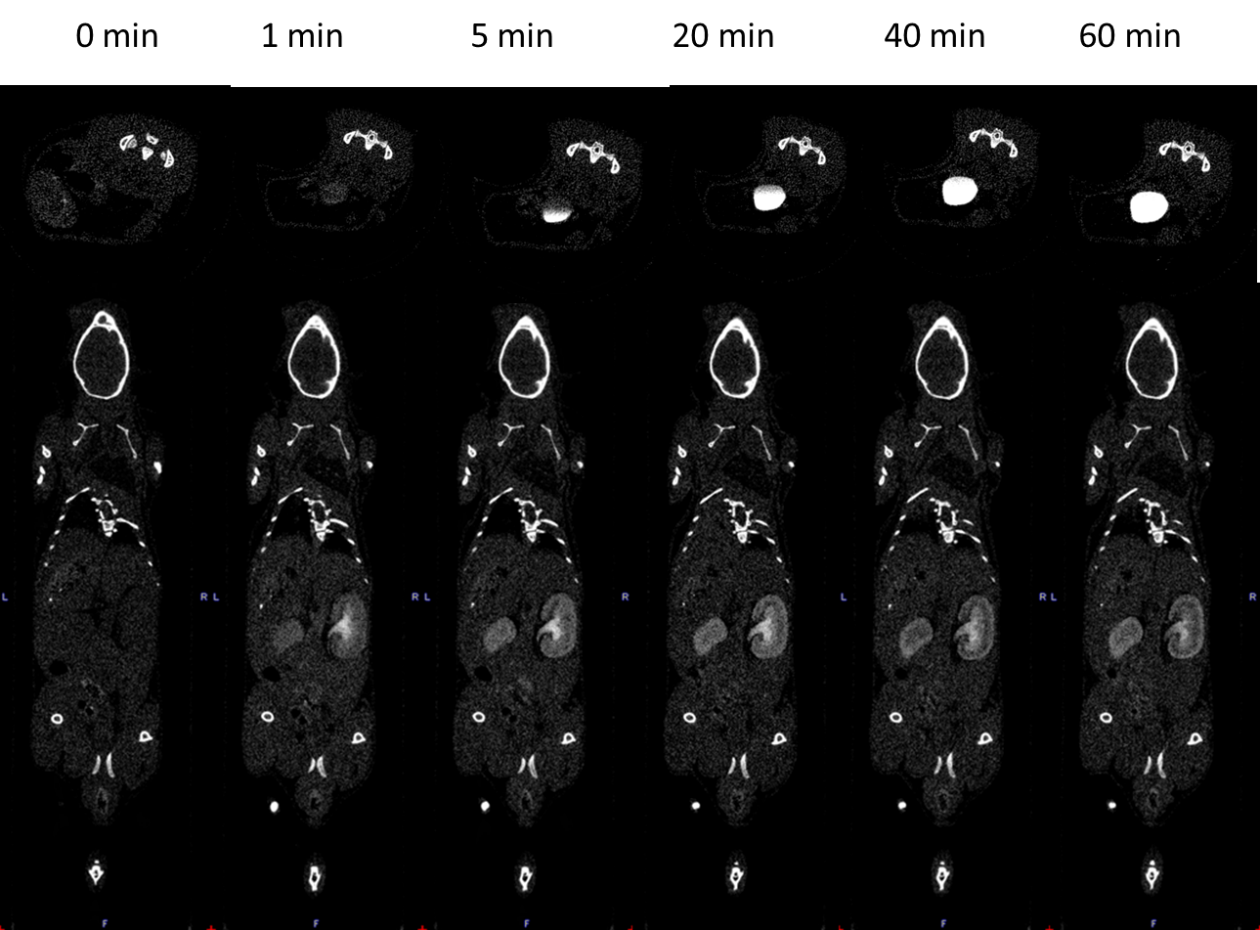
**Figure S8 (a)** *In vivo* CT imaging after i.v. administration of 1.2 mmol/kg of iopamidol. Bladder and kidneys CT contrast are displayed. **(b)** VOIs analysis on bladder and kidneys for a dose of 1.2 mmol/kg of iopamidol.


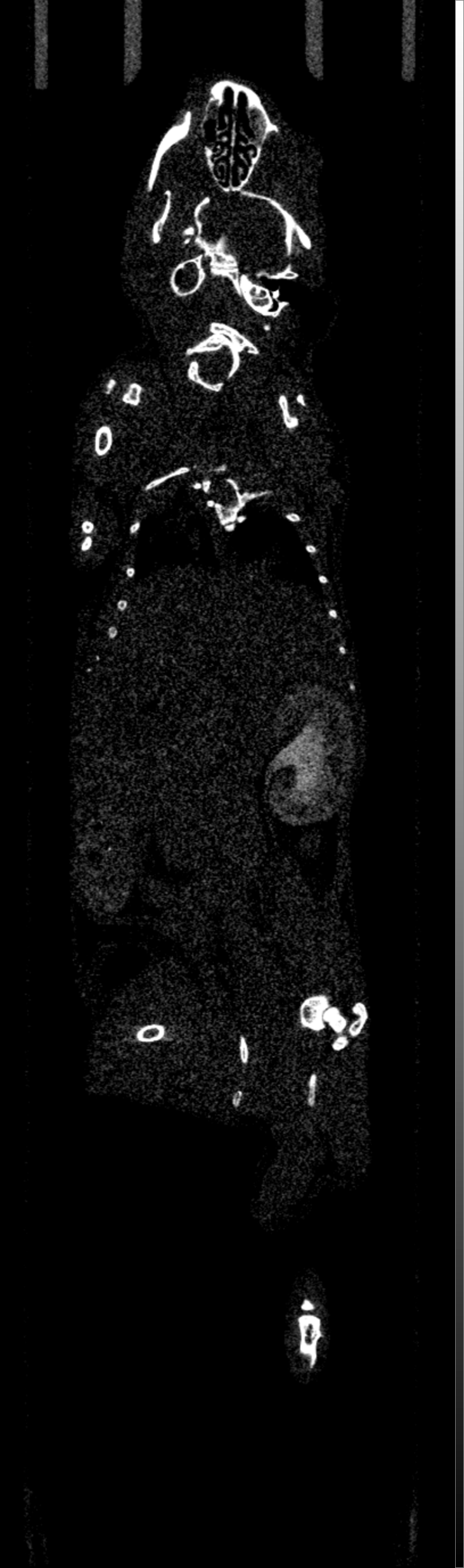


**1500**

**HU**

**0**

**b**

**a**


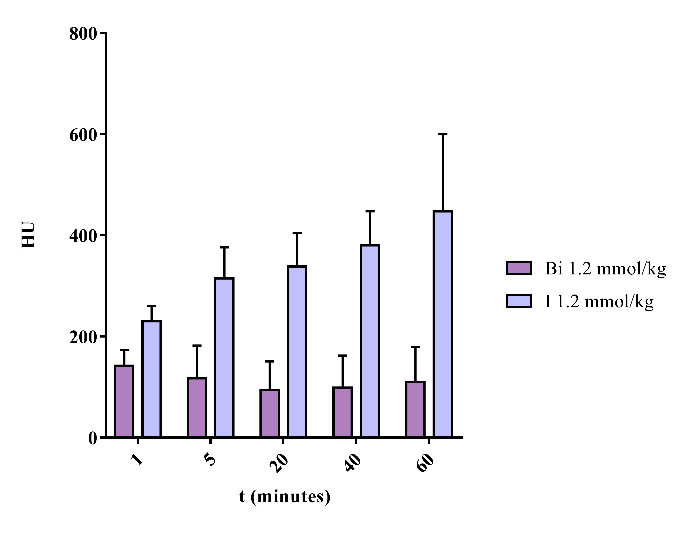


**Figure S9** VOIs comparison analysis in the kidneys after *i.v.* administration of 1.2 mmol/kg of Bi-HPDO3A (purple) or Iopamidol (lilac).


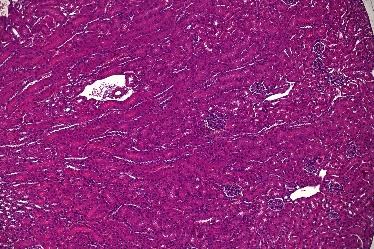

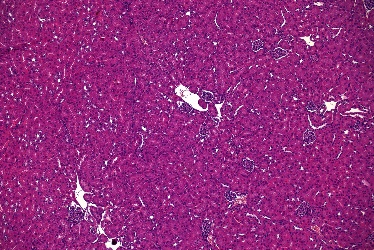

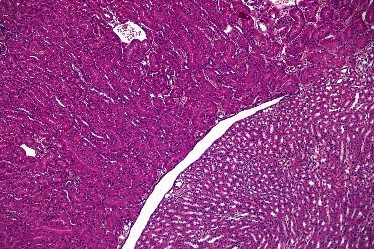


control

5 mmol/kg

7 days

5 mmol/kg

14 days

**Figure S10** HE staining of kidneys after intravenous administration of 5 mmol/kg of Bi-HPO3A after different time points (7 and 14 days) (10x).


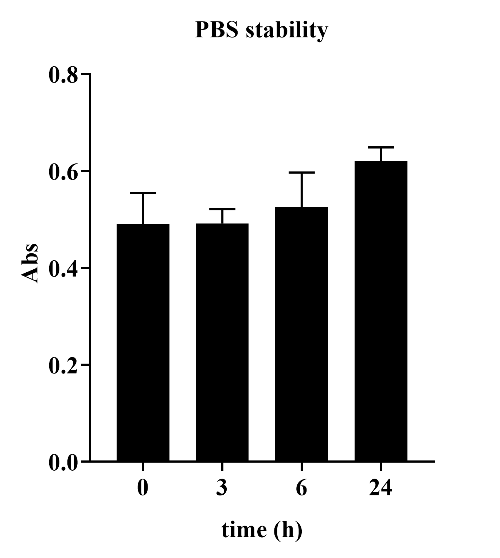

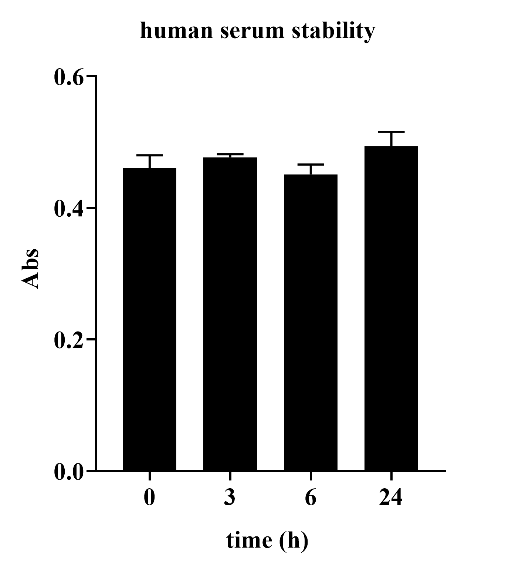


**Figure S11** Stability in HS and PBS of Bi-HPDO3A 0.123M at 37°C within 24h.
